# New Insights into the Diverse Electronic Phases of a Novel Vanadium Dioxide Polymorph: A Terahertz Spectroscopy Study

**DOI:** 10.1038/srep09182

**Published:** 2015-03-17

**Authors:** James Lourembam, Amar Srivastava, Chan La-o-vorakiat, H. Rotella, T. Venkatesan, Elbert E. M. Chia

**Affiliations:** 1 Division of Physics and Applied Physics, School of Physical and Mathematical Sciences, Nanyang Technological University, Singapore 637371, Singapore; 2 NUSNNI-Nanocore, National University of Singapore, Singapore 117411, Singapore; 3 Department of Physics, National University of Singapore, Singapore 117542, Singapore; 4 Singapore Synchrotron Light Source, National University of Singapore, 5 Research Link, Singapore 117603; 5 Department of Electrical and Computer Engineering, National University of Singapore, Singapore 117576, Singapore

## Abstract

A remarkable feature of vanadium dioxide is that it can be synthesized in a number of polymorphs. The conductivity mechanism in the metastable layered polymorph VO_2_(*B*) thin films has been investigated by terahertz time-domain spectroscopy (THz-TDS). In VO_2_(*B*), a critical temperature of 240 K marks the appearance of a non-zero Drude term in the observed complex conductivity, indicating the evolution from a pure insulating state towards a metallic state. In contrast, the THz conductivity of the well-known VO_2_(*M*1) is well fitted only by a modification of the Drude model to include backscattering. We also identified two different THz conductivity regimes separated by temperature in these two polymorphs. The electronic phase diagram is constructed, revealing that the width and onset of the metal-insulator transition in the *B* phase develop differently from the *M*1 phase.

Multi-polymorphic materials – compounds that can assume numerous, different crystal symmetries with the same chemical composition, show great promise as future electronic materials. This is because their structural diversity can give rise to a variety of electrical and optical responses that can be tuned for technological applications such as optical switches, batteries, solar cells, optical filters, spintronic devices, memory devices etc^1^,^2^,^3^,^4^. Vanadium dioxide is one of the most popular polymorphic materials that can assume several crystallographic structures^5^,^6^,^7^. Amongst the several stable and metastable VO_2_ polymorphs, monoclinic VO_2_(*B*) and monoclinic VO_2_(*M*1) are particularly interesting, as these materials display more than thousand-fold changes in conductivity with temperature^8^,^9^. Understanding polymorphism in vanadium dioxide has tremendous practical importance in the design and control of electro-optic materials for future technologies.

The metastable monoclinic VO_2_(*B*) adopts a structure derived from V_2_O_5_ and belongs to the space group *C2/m* (12) compared to the well-known VO_2_(*M*1), which takes up the space group *P2_1_/c* (14)^10^,^11^,^12^. The crystal structure of VO_2_(B) is illustrated in Figure 1(a). A salient structural difference between the monoclinic phases in VO_2_(*B*) and VO_2_(*M*1) is that VO_2_(*B*) has a smaller *β* inter-axial angle^11^ (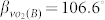 and 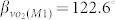) which may play an important role in the formation of defects and impurities in epitaxial growth of films. While metal-insulator transition (MIT) in VO_2_(*M*1) is associated with a corresponding reversible structural phase transition from a room temperature monoclinic to a high temperature tetragonal phase^9^,^13^,^14^,^15^,^16^, currently there is little understanding of the nature of the semimetal-to-insulator transition in VO_2_(*B*) which remains monoclinic with MIT^10^. It is also interesting to compare these polymorphs in context of V-V pairing mechanism, which strongly influences the conductivity pathways. VO_2_(*M*1) undergoes V-V dimerization in the monoclinic insulating phase with very short V-V bonds (~2.65 Å) creating a large dimer-to-dimer distance (~3.12 Å)^14^. This alternate long and short V-V separation inhibits conductivity. But when VO_2_(*M*1) undergoes a phase transition to the rutile structure all the V-V distances become the same (~2.87 Å) and do not allow the formation of dimers^14^. In contrast, one doesn't see a significant change of V-V distance in the *B* phase^10^. One could plainly see that, even though these VO_2_ polymorphs have the same chemical stoichiometry, their structures and electronic attributes are evidently distinct and a comparative study between these polymorphs is aptly motivated by an objective to get a better understanding on the correlation between MIT and structural phase transition. Furthermore, while a lot of progress has been made towards understanding the physics of metal-insulator transition in VO_2_(*M*1) by optical techniques^17^,^18^,^19^,^20^,^21^,^22^,^23^,^24^,^25^,^26^,^27^,^28^, no parallel efforts has been made so far to understand the mechanism in VO_2_(*B*). From a practical applications point of view, one of the key attractions of VO_2_(*B*) is that it is conducting at room temperature, compared to VO_2_(*M*1) which is insulating and may hold technological merits over VO_2_ (*M*1) for devices at room temperature operation^8^.

The drastic changes in conductivity with temperature for these VO_2_ polymorphs are accompanied by a corresponding change in optical response, thereby making terahertz (THz) spectroscopy a viable technique to probe the nature of electronic properties in these systems. There have been several reports where THz spectroscopy has been employed to measure conductivity in VO_2_(*M*1) polymorphs^25^,^26^,^29^,^30^,^31^. Although metal-insulator transition materials are generally characterized by standard four-point probe method, it provides information only about dc conductivities which can be distorted by surface defects due to electrode contacts^32^. On the other hand, THz spectroscopy is a contactless probe, providing frequency-dependent complex conductivity in the far-infrared region. Terahertz time-domain spectroscopy (THz-TDS) allows the amplitude and phase of the THz pulse to be obtained, without the need for Kramers-Kronig analysis in the extraction of complex conductivity^33^,^34^,^35^. Moreover, compared to traditional steady-state dc conductivity measurements which is limited to macroscopic conductivity paths, THz spectroscopy explores electron dynamics over nanometer length scales, possibly revealing conducting phases in this scale undetectable by long-range transport measurements^29^. Indeed, THz spectroscopy has been used to make comprehensive optical investigations in a host of materials rich in exotic conducting phases including graphene^36^,^37^, toplogical insulators^34^,^38^, superconductors^39^,^40^,^41^, oxide semiconductors^32^,^33^, quantum-confined semiconductors^42^, and percolating systems^43^.

In this paper we report a systematic investigation of THz complex conductivities of 60-nm thick VO_2_(*B*) thin films deposited on (001) SrLaAlO_4_ (SLAO) substrate. There has been no literature report on the complex conductivities of this system. In our operational THz frequency range (0.3–2.3 THz), there are no observable optical phonon resonances of VO_2_(*B*), and hence it is possible to observe the conductivity response that arises solely from free carriers. These are then compared with that of 60-nm VO_2_(*M*1) thin films deposited on SLAO substrate. This study conclusively demonstrates clear signatures of different electronic orders in these two vanadium dioxide polymorphs.

## Results

### Sample growth, structural and transport characterization

**Figure 1:**
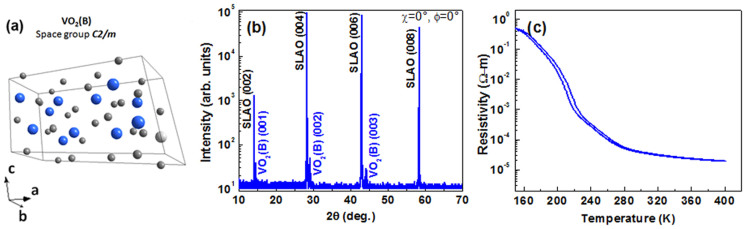
(a) Crystal structure of the VO_2_(*B*) polymorph. The vanadium atoms are represented by blue spheres and the oxygen atoms are represented by dark grey spheres.(b) XRD θ–2θ pattern for the VO_2_(*B*) film epitaxially grown on (001) SLAO substrate. (c) Temperature dependent resistivity of VO_2_(*B*) on SLAO measured using a traditional four point geometry.

The XRD spectra of VO_2_(*B*) thin film on SLAO is shown in Figure 1(b). We clearly see that the XRD reflections of VO_2_(*B*) are perfectly aligned with the substrate (azimuthal angle, χ = 0). This means that our film is highly oriented. Temperature-dependent electrical transport measurements are carried out in Physical Property Measurement system (Quantum Design) utilizing a four probe geometry. As shown in Figure 1(c), the resistivity of VO_2_(*B*) films grown on SLAO substrate undergo four orders-of-magnitude change (from 2 × 10^−5^ Ω-m to 0.35 Ω-m) in resistivity as the temperature changes from 400 K to 160 K, which is consistent with a previous report on VO_2_(*B*) nanorods^8^. There is a weak thermal hysteresis at low temperatures below 240 K.

### THz-TDS

**Figure 2:**
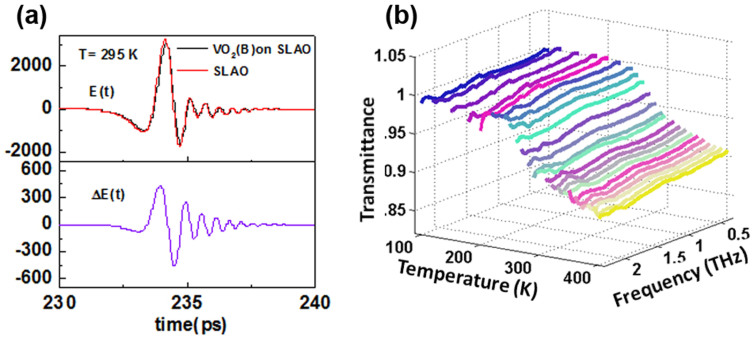
(a) THz signals transmitted through VO_2_(*B*) film deposited on SLAO substrate compared to the transmitted signal through bare substrate at 295 K shown along with monoclinic VO_2_(*B*) cell. While the top panel describes the raw transmitted THz electric-field waveform *E(t)* the bottom panel represents the corresponding difference in the THz waveform *ΔE(t)* between the sample and the reference. (b) Evolution of the transmission spectra with temperature and frequency of VO_2_(*B*) thin film deposited on SLAO substrates during warming process.

Figure 2(a) shows time-domain signal of the main THz pulse [*E(t)*] transmitted through the film and reference for VO_2_(*B*) thin film. After the main pulse, a weaker pulse (etalon pulse) appears due to multiple reflections in the substrate. The main THz pulse and etalon pulse are well separated in the time domain, allowing easy truncation of the time-domain data to remove the etalon pulse. All our subsequent data analysis is done on the main pulse and removal of etalon pulses does not lead to any relevant loss of information. The lower panel of Figure 2(a) is the THz absorption at 295 K by plotting the difference in the electric field time domain signal between the sample and the reference [*ΔE(t)*]. Fast Fourier Transform (FFT) is then performed on the time domain THz signals to obtain the amplitude and phase of the THz transmission spectra.

The transmittance, *T*(*ω*) of the VO_2_ film is defined as the ratio between complex electric field of the THz pulse from sample (film + substrate) 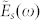 and reference (bare substrate) 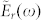. In Figure 2(b) the frequency-dependent and temperature-dependent transmittance amplitudes through the VO_2_(*B*) film are shown. In the insulating state, at temperatures lower than the transition temperature, we find that the transmittance amplitude is close to unity. For VO_2_(*B*) film, as the temperature of the sample approaches ~240 K, there is THz absorption and a clear trend of increasing absorption at higher temperatures. The spectral shape is almost frequency independent for all runs. The frequency dependent complex refractive index 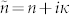 can be extracted from the experimental transmittance *T*(*ω*) by fitting the formula^36^,^44^
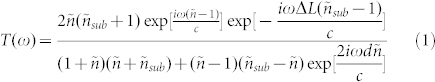
where 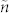 and 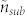 are the complex refractive indices of VO_2_ film and SLAO substrate, respectively, *d* is film thickness and Δ*L* is the thickness difference between sample and reference substrates and *c* is the speed of light in vacuum. This above equation takes into account the multiple internal reflections inside the film.

The extracted complex refractive index 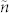 is then used to calculate the complex optical conductivity 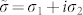 where *σ*_1_(*ω*) = 2*nκωε*_0_ and *σ*_2_(*ω*) = (*ε*_∞_ − *n*^2^ + *κ*^2^)*ωε*_0_ where *ε*_0_ is the permittivity of free space and *ε*_∞_ is the high frequency dielectric constant. For VO_2_(*B*), *ε*_∞_ is an unknown quantity and is initially set to 1. After the determination of complex conductivities, *ε*_∞_ will be used as a temperature-dependent fitting parameter in the conductivity models as discussed in the following sub-sections. On the other hand, we used *ε*_∞_ = 9 based on previous studies for determining conductivities in VO_2_(*M*1)^45^,^46^.

### Complex conductivity of VO_2_(*B*)

**Figure 3:**
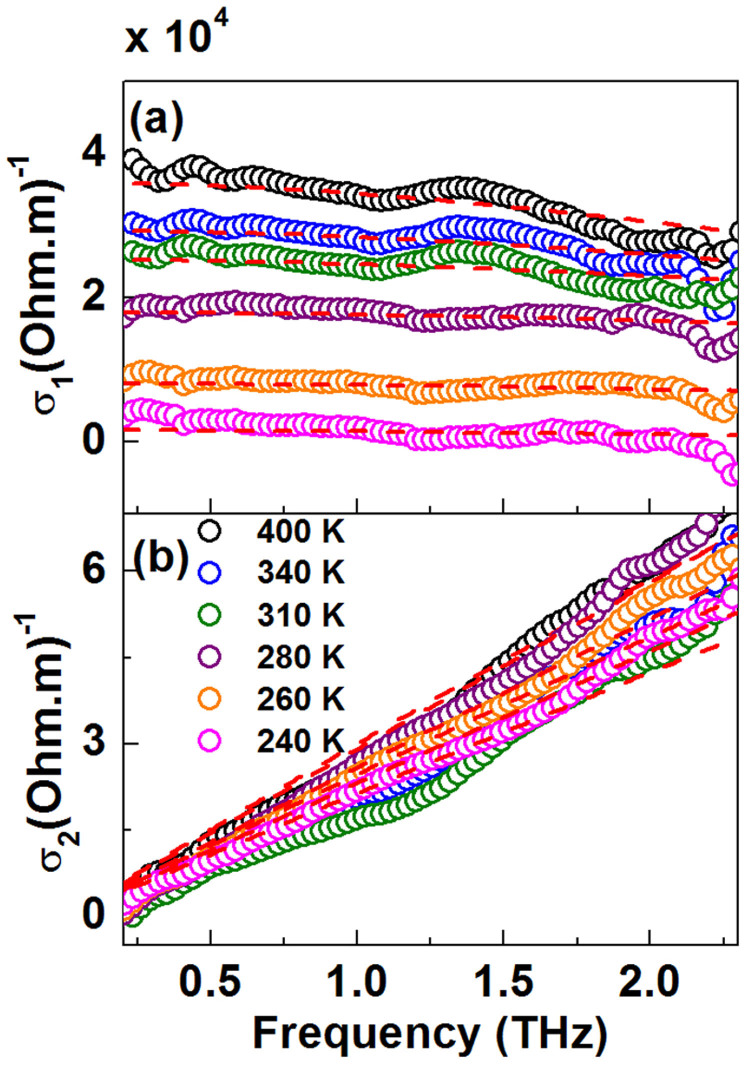
(a) Real and (b) imaginary conductivities in the THz frequency range of VO_2_(*B*) thin films grown on SLAO with fits to the Drude model (red dash lines) shown for temperatures ranging from 240 to 400 K.

The complex optical conductivities of the VO_2_(*B*) films for temperatures ranging from 240 K to 400 K are shown in Figures 3(a) and (b) as a function of frequency during the warming process (open symbols). Below 240 K, the transmission is ~100%, giving zero real conductivity, indicating that the films are clearly insulating. The onset of a positive *σ*_1_(*ω*) marks the transition from an insulating regime to a conducting one. We clearly observe *σ*_1_(*ω*) to increase with increasing temperature throughout our frequency range. Thermal excitation of electrons into the conduction band can account for this nature, that is, a Drude response where *σ*_1_(*ω*) is a maximum at low frequency and decreases with increasing frequency, while *σ*_2_(*ω*) are zero at low frequency and increases with increasing frequency.

**Figure 4:**
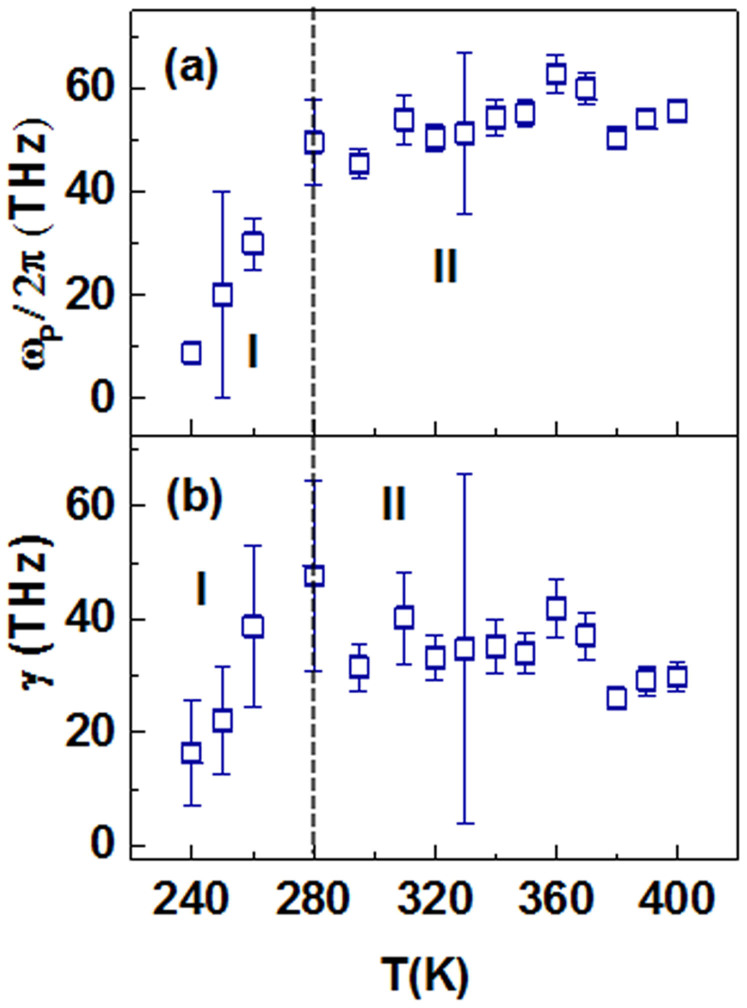
Temperature dependence of (a) the plasma frequency and (b) the scattering rate, for *T* ≥ 240 K as estimated from the Drude fitting. The dashed lines are guide to the eye demarcating the two different electronic orders.

According to the Drude model, the frequency dependent complex optical conductivity is given by
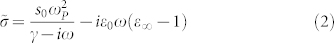
where *ω_P_* is the Drude plasma frequency, *γ* is the free carrier scattering rate and *ε*_∞_ is the high frequency dielectric constant as defined earlier. Both *σ*_1_(*ω*)and *σ*_2_(*ω*) at each temperature measurement are simultaneously fitted to Equation 2, as shown by the dash red lines in Figures 3(a) and (b) respectively. The fits are reasonably good for all temperatures. The fittings results of the Drude parameters are shown in Figures 4(a) and (b). The simultaneous fitting of *σ*_1_(*ω*) and *σ*_2_(*ω*) to the Drude model greatly reduces the width of the error bars of the fitted *γ* and *ω_P_* even though *σ*_1_(*ω*) is relatively featureless.

We observe a similar trend in both the plasma frequency and the scattering rate – both parameters increases as we increase the temperature up to ~280 K, and tends to saturate beyond 280 K. This common trend in *ω_P_* and *γ* describe two different temperature-dependent conductivity mechanisms. To address this issue and to make a distinction, we describe the temperature regions 240 K ≤ *T* ≤ 280 K and 280 K <*T* ≤ 400 K as conductivity regimes I and II respectively.

In regime I, *ω_P_*/2*π* rises from (8 ± 1) THz to (49 ± 8) THz, while in regime II this parameter has a mean value of ~ (54 ± 5) THz. The plasma frequency is closely related to the number of charge carriers (*N*) in the system via 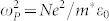 where m* is the effective mass. The scattering rate, (*γ*) is associated with the mobility of the carriers, (*μ*) via the expression *γ* = *e*/(m*μ). Without the knowledge of the carriers' effective mass, accurate values of *N* and *μ* in VO_2_(*B*) cannot be determined. In the later sub-sections, more discussions regarding the nature of m* will be presented.

**Figure 5:**
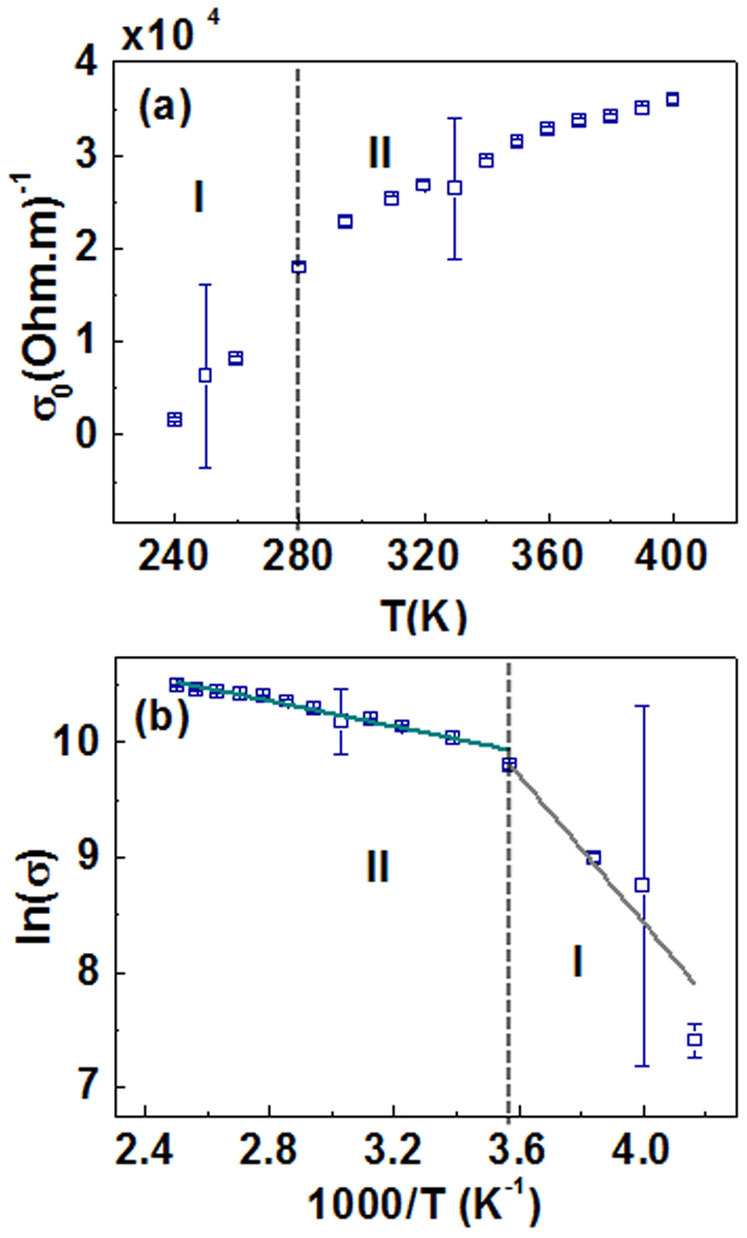
(a) Temperature dependence of dc conductivity of VO_2_(*B*) films on SLAO as estimated from Equation 3. (b) Logarithmic plot of conductivity ln(*σ*) vs reciprocal temperature 1000/T shown alongside linear fitting and the conductivity regimes I and II.

The Boltzmann dc conductivity *σ*_0_ can be obtained from the expression
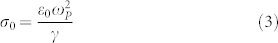
Figure 5(a) shows the calculated dc conductivity using Equation 3. The plot shows *σ*_0_ increasing with increasing temperatures, but the rate of change is significantly smaller at higher frequencies. We recall that while *ω_P_* and *γ* start saturating at 280 K, *σ*_0_ does not. However, for *T* > 280 K and *T* ≤ 280 K, *σ*_0_ changes with different slopes and hence can be correlated with our description of conductivity regimes I and II. If we compare, our estimated dc conductivity from the THz measurements are consistent with the electrical transport measurements shown in Figure 1(c). Figure 5(b) shows the logarithmic conductivity, ln(*σ*) plotted against reciprocal temperature, 1000/*T*. The activation energy, *E_a_* can be estimated from the thermal activation model of dc conductivity which has the following temperature dependence
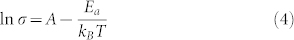
where *k*_B_ is the Boltzmann constant. The linear fittings of the conductivity regimes with the activation model show a good agreement. Fits to Equation 4 yields *E_a_* (regime I) = (260 ± 30) meV, and *E_a_* (regime II) = (47 ± 3) meV. This significant difference in activation energies supports our claim that there are two different temperature dependent conductivity mechanisms in VO_2_(*B*). In regime I, the conductivity mechanism is poor with larger activation energy, while in regime II, the conductivity is better with smaller activation energy. The presence of small activation energy in conductivity regime II shows that even at high temperatures it has not achieved a fully metallic state. The features of metal-insulator transition in VO_2_(*B*) can be understood as follows: (i) Temperatures below 200 K can be identified as a pure insulating state displaying 100% transmission in the insulating state, (ii) conducting state I is an intermediate electronic phase transition characterized by strong dependence of Drude parameters with temperature, (iii) conducting state II describes the phase after the transition is completed. The boundaries of various electronic phases driven by temperature are thus more clearly defined in the THz-TDS study compared to the electrical transport measurements where the change in resistivity is gradual and not well discernible.

### Comparison with VO_2_(*M*1)

**Figure 6:**
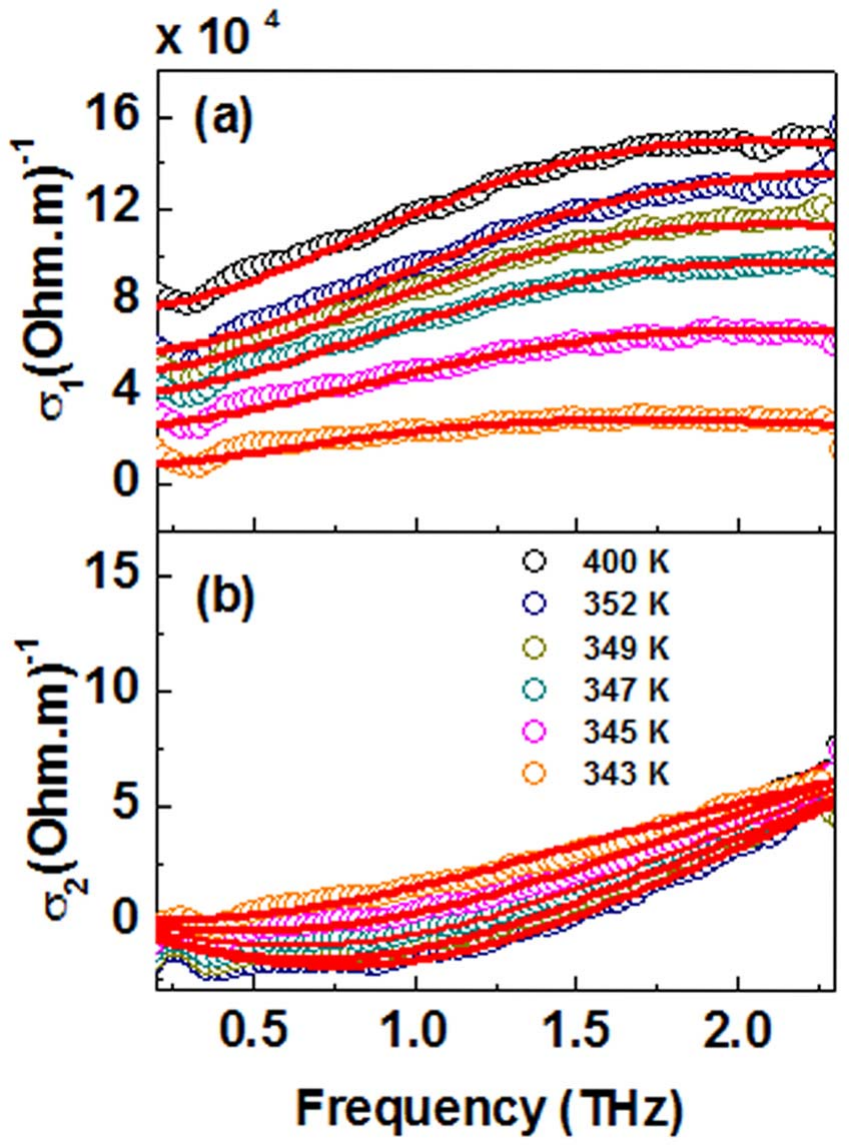
(a) Real and (b) imaginary conductivities of VO_2_(*M*1) films on SLAO with fits to the Drude-Smith model (sold red lines) for six selected temperature points in the range 343 K–400 K during the warming process.

The THz conductivity spectra of 60-nm thick VO_2_(*M*1) film grown on SLAO are shown in Figure 6 plotted as the temperature of the system is gradually increased from 343 K to 400 K. In contrast to VO_2_(*B*), throughout this temperature range, *σ*_1_(*ω*) increases with increasing frequency, while *σ*_2_(*ω*) starts at zero at low frequencies, becomes negative, then crosses over to positive with increasing frequency. This is consistent with the previous reported THz studies of VO_2_(*M*1) films^25^,^26^,^30^.

In classical VO_2_(*M*1) systems it has been reported that the complex conductivities can be effectively modelled by Drude-Smith theory, which is a generalisation of the Drude model proposed by Smith, in order to account for the conductivity suppression due to charge localization^33^,^43^,^47^,^48^. In this polymorph, it is widely considered that the transition from a highly transparent insulating phase to an absorbing metallic phase occurs through a percolating network^29^,^46^,^49^. Near the threshold of this transition, phase separation and domain formation dictate the electronic pathways. The frequency-dependent optical conductivity in the Drude-Smith model is given by^47^
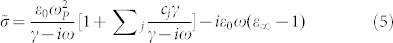


The additional terms described by *c_j_* represents the fraction of the carrier's initial velocity retained after *j* number of scattering events^47^. In various systems such as poor conductors, metal-insulator transition materials and various nanomaterials where the Drude-Smith model is successfully applied, only the first scattering term is considered (i.e. *j* = 1)^30^,^48^,^50^,^51^,^52^. In this scenario, carrier transport transitions from ballistic to diffusive after one scattering event, leading to complete momentum randomization^47^. In our fitting analysis also we will only consider the first *c_j_* term and in this case we take *c*_1_ = *c*. The parameter *c* can take up any values anywhere between 0 (free Drude carrier conduction) and −1 (full carrier backscattering)^43^. There have been other reports of using effective medium theory (EMT) models to fit THz conductivities of VO_2_(*M*1) with varying degrees of success^29^,^31^. Our attempts to model the optical conductivities using the effective medium models gave inconsistent results (Supplementary Information). On the other hand, as shown by the fitting lines in the Figures 6 (a) and (b), the Drude-Smith formalism given by Equation (5) when *j* = 1 gives excellent fits to our measured conductivities of *σ*_1_(*ω*) and *σ*_2_(*ω*). We could see that even at high temperatures the conductivity does not follow an ideal Drude behaviour which indicates that the nanogranular boundaries persist even after the transition. In highly granular systems, the Drude-Smith is much more effective in describing the effective conductivity rather than a conventional EMT models because it can describe effective conductivities of systems where grain boundaries persist even in large conducting fractions^30^,^43^. The advantage of Drude-Smith over EMT models in this scenario has been discussed by T. L. Cocker *et.al*^30^.

**Figure 7:**
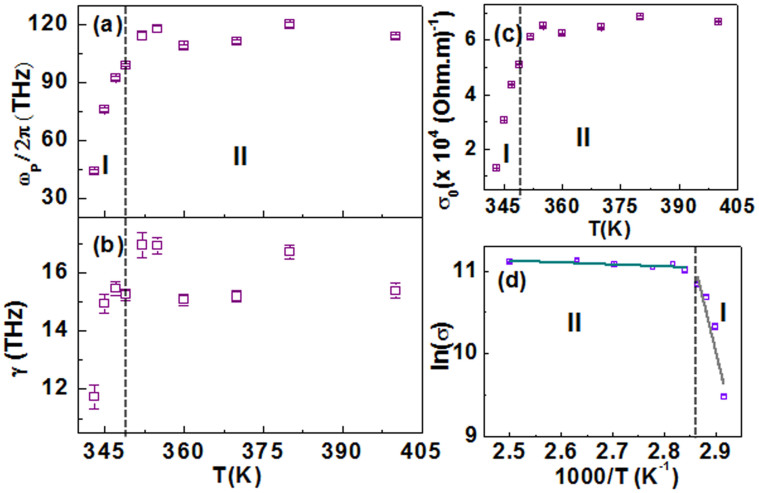
Temperature dependence of Drude-Smith fitting parameters: (a) the plasma frequency, *ω_P_*/2*π* (b) the scattering rate, *γ* for *T* ≥ 343 K and (c) dc conductivity as estimated from Equation 3 for VO_2_(*M*1) films grown on SLAO during the warming cycle. The dashed lines separates the conductivity regimes I and II. (d) Logarithmic conductivity, ln(*σ*) dependence on reciprocal temperature 1000/*T* is shown and fitted with a linear relation.

Simultaneous complex fitting of optical conductivities gives the information about the parameters *ω_P_*, *γ* and *c*. Figure 7 summarizes the *ω_P_*, *γ* parameters obtained from the fitting procedure as well as the dc conductivity *σ*_0_ which is given by the equation, 
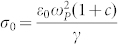
. The Drude-Smith parameters *ω_P_* and *γ* increase with increasing temperatures in the temperature range 343 K–349 K; above 349 K they remain largely temperature-independent. This characteristic is similar to the temperature dependence of Drude parameters in VO_2_(*B*). In the temperature range 352 K–400 K, the mean value of *ω_P_*/2*π* is ~ (110 ± 4) THz. The fitting parameter *c* lies between −0.88 and −0.75 which indicates significant carrier backscattering – an attribute of carrier localization, resulting in significant suppression of low-frequency conductivities. The temperature dependence of the fitted values of *c* supports the percolation picture in VO_2_(*M*1)^43^.

We find that similar to *ω_P_* and *γ*, *σ*_0_ increases with increasing temperatures till 349 K; at higher temperatures, it attains a mean saturating value of (6.5 ± 0.3) × 10^4^ (Ohm.m)^−1^. Analogous to VO_2_(*B*), we can define the temperature range of 343 K–349 K as conductivity regime I and 352 K–400 K as conductivity regime II. However in VO_2_(*M*1), *σ*_0_ remains largely temperature independent in conductivity regime II, while in VO_2_(*B*) there is a weak temperature dependence in this regime. Figure 7(d) shows the linear fitting plot of logarithmic conductivity, ln(*σ*) versus reciprocal temperature, 1000/*T*. Similar to our previous analysis of VO_2_(*B*), we can determine the activation energy of VO_2_(*M*1) from the slope of the linear relation. For VO_2_(*M*1), in the low-temperature regime I, the activation energy *E_a_* = (2200 ± 500) meV while in the high-temperature state II, *E_a_* = (20 ± 8) meV. The difference between the activation energies in regions I and II is much larger in VO_2_(*M*1) than in VO_2_(*B*) which is consistent with our *ω_P_* and *σ*_0_ results where the quantitative growth of these values as the system goes from regime I to II is much larger in VO_2_(*M*1) compared to VO_2_(*B*). Also, while the activation energies in regime II are similar in these VO_2_ polymorphs, *E_a_* of VO_2_(*M*1) is an order of magnitude larger than that of VO_2_(*B*) in regime I. This means that in regime I, the conductivity slope is steeper in VO_2_(*M*1) and concomitantly we found a sharper rise of *ω_P_* and *γ* in VO_2_(*M*1) in regime I.

According to M. M. Qazilbash *et. al.*^22^, a significant enhancement of the carrier effective mass, m* (as large as 5*m_e_*, where *m_e_* is mass of an electron) is found in the infrared spectra, especially in the intermediary phase transition state which is analogous to our conductivity regime I. After the transition is completed (at *T* ~ 350 K), m* is close to 2*m_e_* which is the value reported by other experiments^46^. Considering m* = 2*m_e_*, at temperatures just above the transition (*T* = 352 K), we obtain the carrier density to be ~3.6 × 10^20^ cm^−3^ from the plasma frequency. In the case of Drude-Smith model, the relationship between *γ* and *μ* is modified as, 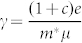. This relationship gives *μ* = 11.35 cm^2^/(V·s) at *T* = 352 K in VO_2_(*M*1). Previous THz-TDS experiments on VO_2_(*M*1) reported similar values of carrier density and mobility^30^,^31^. Hall effect measurements reported similar values of carrier density at this temperature but the reported mobility is lower, which is probable as THz study does not face the challenges in Hall measurements due to low-mobility^53^. The fundamental difference between these two techniques is that while Hall measurement is meant for DC conductivity measurements THz-TDS is an AC measurement method. Hall measurement being a contact method requires the charge carriers to travel substantial distance across the biased electrodes but on the other hand THz-TDS captures the conductivity traversed in sub-pico second timescales resulting in ‘conductivity snap-shots'^42^. As a consequence, THz-TDS can give conductivity information before the carriers are trapped or recombined and as such it is quite reasonable to expect that the THz mobility would be higher than the Hall mobility. We can also compare the carrier density and the mobility parameters of VO_2_(*M*1) with that of VO_2_(*B*). By assuming m* = 2*m_e_* at *T* = 295 K for VO_2_(*B*), which is just after the transition, the calculated values are *N* ≈ 6.4 × 10^19^ cm^−3^ and *μ* ≈ 21.9 cm^2^/(V·s).

It has been reported that the first-order phase transition in VO_2_(*M*1) takes place via percolation where metallic domains embedded in a matrix of insulating phase starts nucleating sporadically as temperature is increased. When the temperature reaches the metal-insulator transition temperature *T_MI_*, the domains become well connected to form a percolating path in this inhomogeneous composite medium. The Drude-Smith model has been applied to various percolating systems where inhomogeneity in conductivity contributes of a non-zero backscattering parameter *c*. On the other hand VO_2_(*B*) can be described by a simple Drude model, although it is a poor conductor in comparison to VO_2_(*R*). The fact that VO_2_(*R*) cannot have an ideal Drude behavior can be explained by its granular origin, intrinsic inhomogeneity. In VO_2_(*B*) we cannot see evidence of inhomogeneous phase separation or percolating transport from the THz study and thus shows a good Drude fit. The explanation for this difference between the polymorphs is two-fold — first, VO_2_(*B*) is a more uniform film in the context of epitaxial growth with lesser roughness and hence no backscattering. VO_2_(*M*1) on the other hand, has a more grainy morphology due to the larger *β* inter-axial angle (Supplementary Information). Second, the activation energy is larger in VO_2_(*B*) in the conducting state and so the number of thermally activated carriers are smaller.

**Figure 8:**
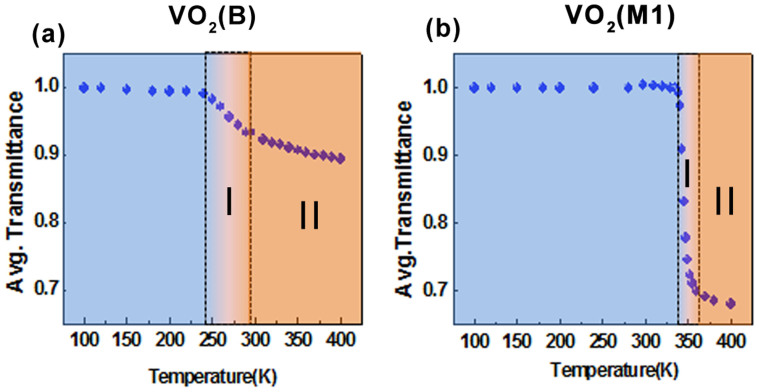
Normalized and frequency-averaged THz transmission of (a) VO_2_(*B*) and (b) VO_2_(*M*1) thin films as a function of temperature during the warming process. These values are normalised to the transmission at 100 K. The blue colour regions represent the insulating phases while the pure conducting phases are coloured orange. The two dotted lines mark the intermediate transition state. The conductivity regimes I and II as described in the previous discussions are also labelled.

Furthermore, the narrow intermediate state of VO_2_(*M*1), which falls in our conductivity regime I, is a strongly correlated phase marked by enhanced mass and an optical pseudo-gap before crossing over to a rutile metallic state as indicated by several optical measurements including infrared spectroscopy, ellipsometry and femtosecond pump-probe measurements^22^,^23^,^49^,^54^. It is also possible that in the intermediate state of VO_2_(*B*), electronic correlations may play a significant role in this intermediate transition state. The rich electronic phase diagram of these vanadium dioxide polymorphs can be summarized from our THz transmission spectra as illustrated in Figure 8.

## Discussion

In conclusion, we have used THz spectroscopy to measure the temperature-dependent complex optical conductivity of vanadium dioxide polymorphs. In our operational frequency range (0.3–2.3) THz, VO_2_(*B*) exhibits no phonon modes and the onset of Drude conductivity behaviour arrives for temperatures higher than 240 K. On the other hand, in VO_2_(*M*1) the THz conductivity can be better described by the Drude-Smith model. Key parameters of carrier dynamics (i) plasma frequency (*ω_P_*) and (ii) scattering rate (*γ*) are obtained by fitting to the Drude (in VO_2_(*B*)) and Drude-Smith in (VO_2_(*M*1)) models. Interestingly, our THz analysis revealed that like in VO_2_(*M*1), VO_2_(*B*) also transforms from an insulating system to a conducting system but is mediated by a much broader intermediate state with the transition onset much closer to room temperature making it more suitable than the widely studied VO_2_(*M*1) for optoelectronic devices operating at *room temperature*. Our study also demonstrates that these polymorphs can also be used as temperature-controlled THz frequency modulators. While the sharp phase transition in VO_2_(*M*1) is suitable for digital-like THz modulation, the broad phase transition in VO_2_(*B*) offers analog-like continuous modulation of THz spectra very similar to W-doped VO_2_(*M*1)^55^. THz conductivity study is a fascinating subject of research from viewpoints of phase transitions in these systems. We show that THz spectroscopy is an excellent method of studying polymorphism in materials.

## Methods

### Sample growth

Pulsed Laser Deposition is used to fabricate the various VO_2_ polymorph thin films by ablating a commercial Vanadium single crystal (100) orientated target on the SLAO (001) substrate. The growth temperature is 500°C and the oxygen pressure is varied from 5 × 10^−3^ to 7 × 10^−3^ Torr. By systematic control of oxygen growth pressure and pulse laser frequency, the various phases of VO_2_ are stabilized. Detailed growth conditions study has been reported in a previous study^56^.

### XRD and AFM

The *θ*–2*θ* diffraction data measurements are done on a Bruker D8 Discover diffractometer CuK_α1_ (λ = 1.54 Å). Temperature-dependent electrical transport measurements are carried out in Physical Property Measurement system (Quantum Design) utilizing a four probe geometry. The surface morphology analysis of the various films are characterized on an atomic force microscope (AFM, Dimension V, Veeco).

### THz-TDS

THz transmission spectra measurements of the VO_2_ films are carried out using the TeraView Spectra 3000 THz-TDS system, incorporated with a Janis ST-100-FTIR cryostat. The operational temperature range of the cryostat is 10 K to 450 K. In this system low temperature-grown GaAs films is used for the generation and detection of THz waves. THz time-domain data of VO_2_ films are taken with respect to the signal transmitted through the bare substrate as a reference. Optical properties of the bare SLAO substrate are determined by THz-TDS, with vacuum as the reference signal. The complex refractive index, 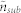 of SLAO substrate is obtained to be 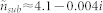 and almost temperature and frequency-independent, and thus is a very suitable THz transparent material in our operational temperature and frequency range. The cryostat is equipped with a vertical motorized stage with a resolution of 2.5 μm which is used to control the back and forth motion between the sample and reference positions.

The aperture diameter used is 7 mm which allowed for an accurate measurement of the THz signal for frequencies as low as 0.3 THz. For each sample and reference run, 900 THz traces are taken in 3 minutes. The thickness difference between the film substrate and the bare substrate (reference), must be taken into account in our subsequent analysis^57^. The sample is kept in a high vacuum (~10^−6^ mbar) for all our experiments.

## Author Contributions

T.V. and E.E.M.C. conceived the project. J.L., T.V. and E.E.M.C. designed the research and E.E.M.C. supervised the project. A.S. fabricated the samples and conducted electrical measurements. H.R. took X.R.D. measurements. J.L. performed the AFM measurements. THz-TDS data were taken by J.L. and C.L. and analyzed by J.L. and E.E.M.C. The manuscript was prepared by J.L. and E.E.M.C. with assistance from T.V., C.L. and A.S. All the authors contributed to discussion on the results for the manuscript.

## Supplementary Material

Supplementary Information Supplementary Information
