# Supplementary material for: New Insights into the Diverse Electronic Phases of a Novel Vanadium Dioxide Polymorph: A Terahertz Spectroscopy Study
Source: Sci Rep. 2015 Mar 17;5:9182. doi: 10.1038/srep09182 (PMC4361872; doi:10.1038/srep09182)
Supplement: Supplementary Information [file srep09182-s1.doc]

**Supplementary Information**

**New Insights into the Diverse Electronic Phases of a Novel Vanadium Dioxide Polymorph: A Terahertz Spectroscopy Study**

James Lourembam ^1^ , Amar Srivastava ^2,3^ , Chan La-o-vorakiat ^1^, H. Rotella ^2, 4^, T.Venkatesan ^2, 3, 5^, Elbert E. M. Chia ^1^

*^1^ Division of Physics and Applied Physics, School of Physical and Mathematical Sciences, Nanyang Technological University, Singapore 637371, Singapore*

*^2^ NUSNNI-Nanocore, National University of Singapore, Singapore 117411, Singapore*

*^3^Department of Physics, National University of Singapore, Singapore 117542, Singapore*

*^4^ Singapore Synchrotron Light Source, National University of Singapore, 5 Research Link, Singapore 117603*

*^5^ Department of Electrical and Computer Engineering, National University of Singapore, Singapore 117576, Singapore*

^*^Email: elbertchia@ntu.edu.sg


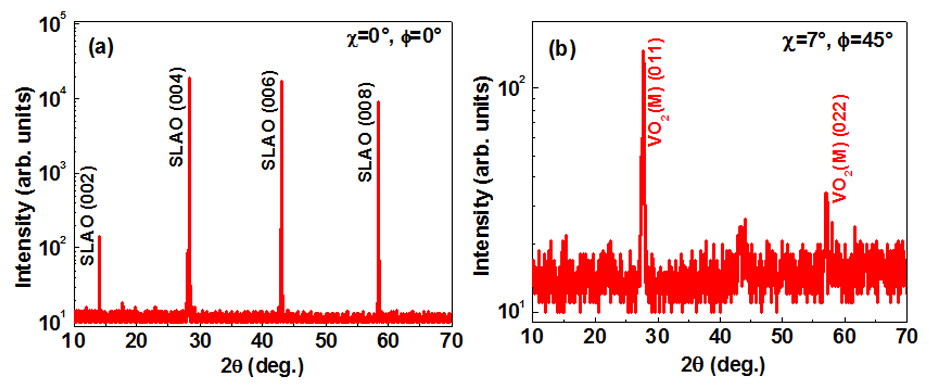


Supplementary Figure 1: XRD patterns for the VO_2_(*M*1) film grown on SLAO taken at different
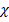
. We can clearly see from (a) that the VO_2_(*M*1) phase reflections are not aligned in
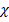
 with SLAO substrate and hence no peaks at
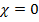
 unlike the VO_2_(*B*) phase film which shows peaks at
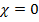
.


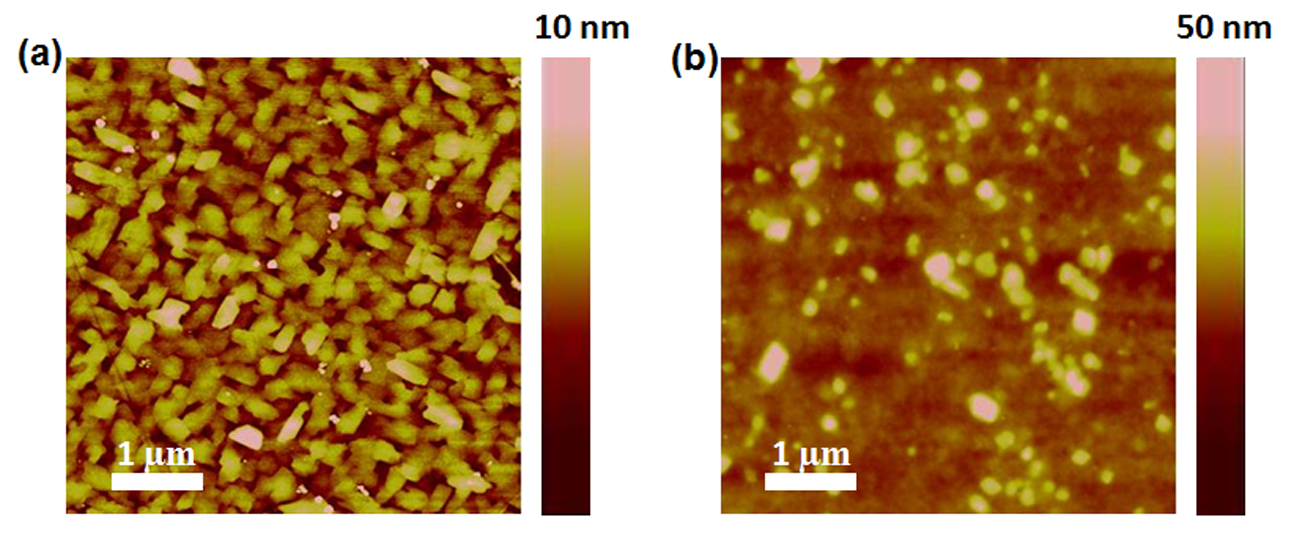


Supplementary Figure 2: Typical AFM image of (a) VO_2_(*B*) and (b) VO_2_(*M*1) grown on SLAO substrates with a scan size of 5×5 μm^2^ for both images. The RMS roughness measured for VO_2_(*B*) and VO_2_(*M*1) are 0.98 and 2.61 nm, respectively.


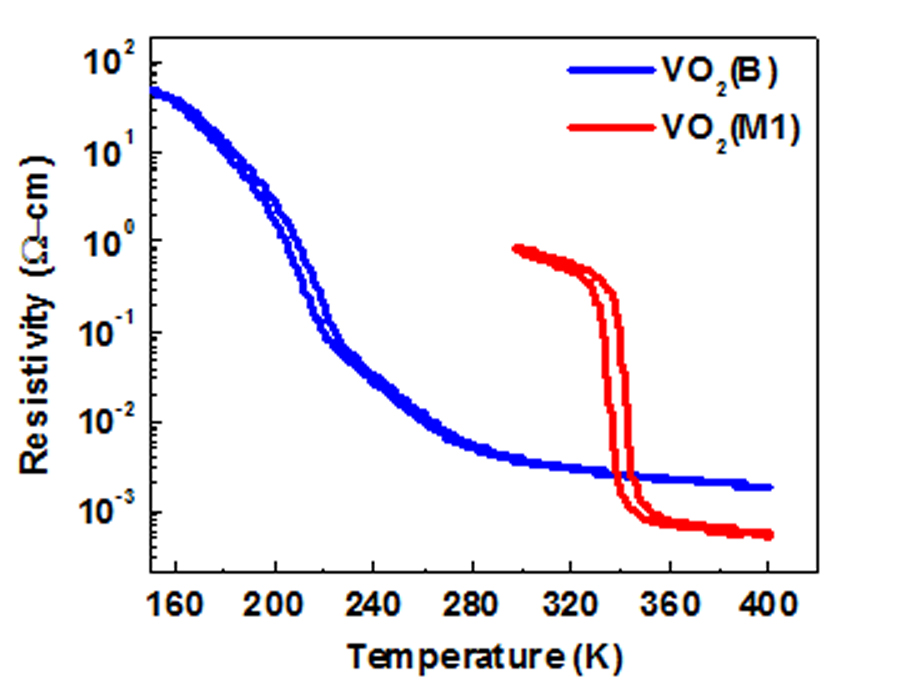


Supplementary Figure 3: Temperature dependent resistivity curves for various vanadium dioxide polymorphs. The electrical transport measurements are done on PPMS Quantum design by adopting four-point probe geometry. The temperature dependent resistivity curves for VO_2_(*M*1) show metal insulator transition at 340 K for the warming process and 330 K for the cooling process which is in agreement with previous reports on this polymorph. Resistivity curves of VO_2_(*B*) films are also re-plotted for comparison. Both films are of 60 nm thickness.


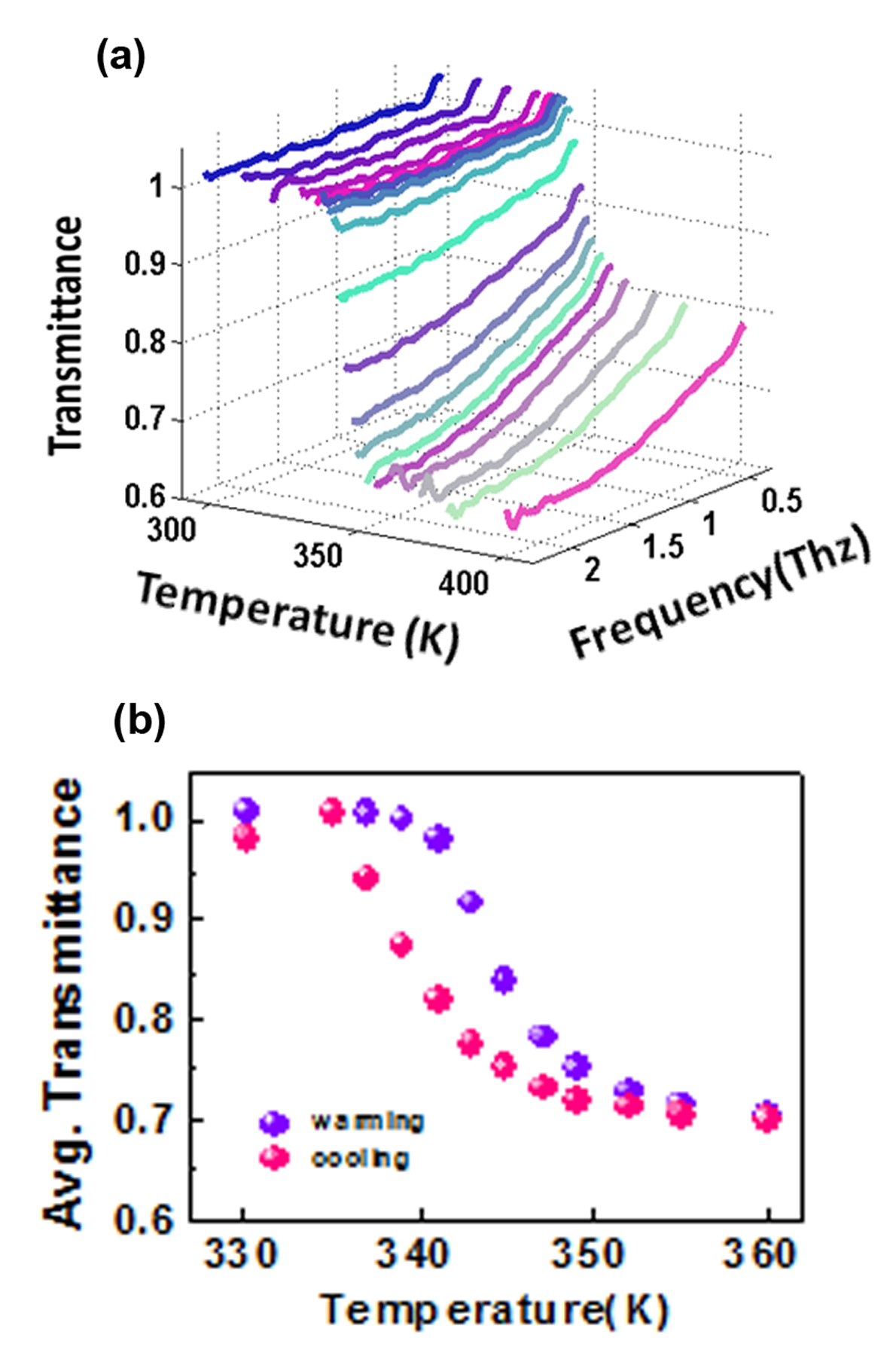


Supplementary Figure 4: (a) Frequency and temperature dependent transmission spectra of 60 nm-thick VO_2_(*M*1) film on SLAO. (b) Normalized transmittance for VO_2_(*M*1) plotted against temperature accompanied by a persistent hysteresis . The onset of absorption is observed at ~340 K and VO_2_(*M*1) undergoes a sharp MIT with a transition width of ~10 K. We also confirm the presence of hysteresis in the normalised transmittance and is a characteristic of first-order transition.


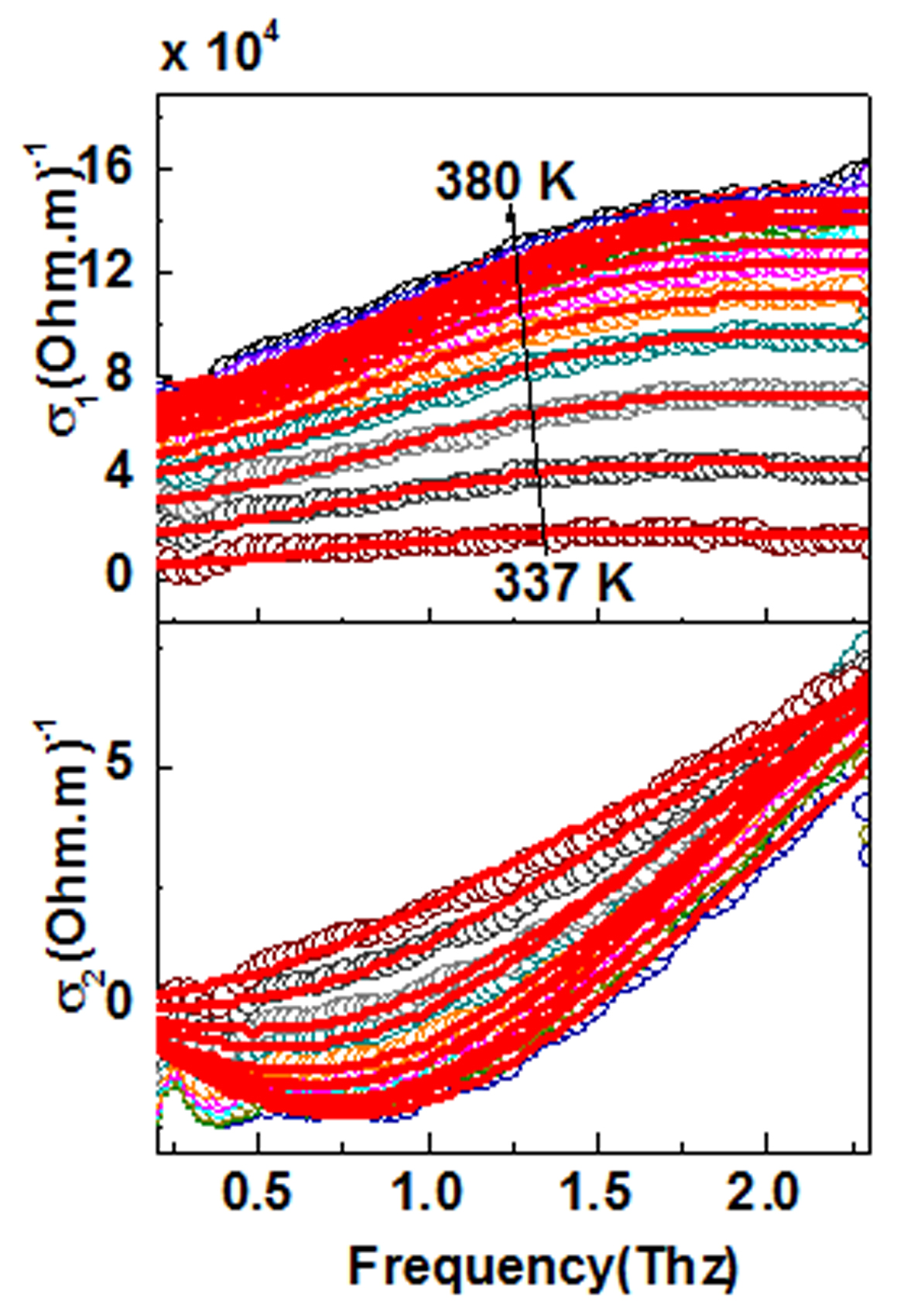


Supplementary Figure 5: Complex conductivities VO_2_(*M*1) film grown on SLAO with Drude-Smith fittings for cooling cycle. The THz conductivity spectra are shown for the temperature scale from 337 K to 380 K. The non-zero values of real conductivity starts appearing at 337 K during the cooling cycle. We recall that this onset of conductivity is observed at 343 K for the warming cycle. The red solid lines represent the simultaneous complex fitting at each temperature. The fitting equation is given by Equation (5)


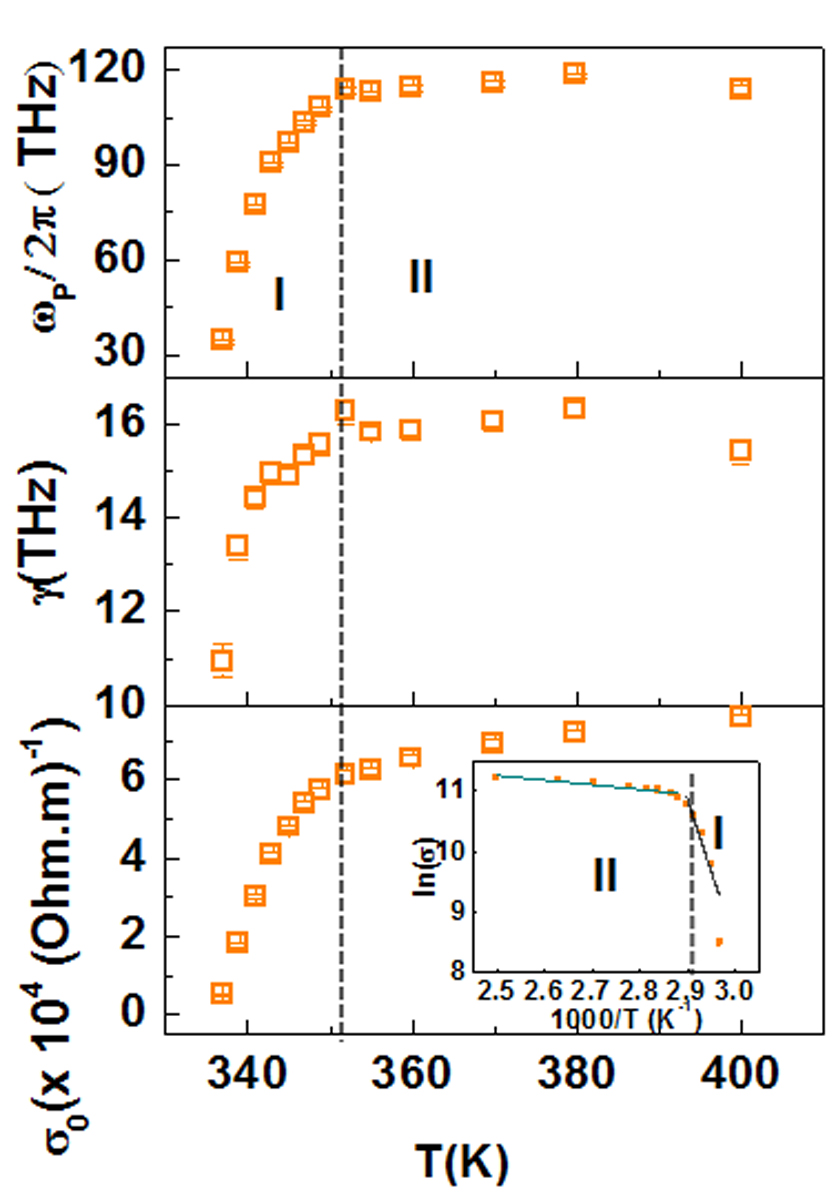


Supplementary Figure 6: Temperature dependence (a) the plasma frequency (b) the scattering rate and (c) dc conductivity for VO_2_(*M*1) films grown on SLAO during the cooling cycle.
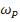
,
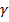
 parameters are obtained from the fitting in Supplementary Fig. 5. The dc conductivity
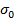
 which is given by the Equation
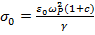
. Inset of (c) shows logarithmic conductivity
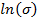
 dependence on reciprocal temperature 1000/T fitted with a linear relation. Similar to the warming cycle, two distinct conductivity regimes on the temperature scale are identified. The activation energies of VO_2_(*M*1) during the cooling cycle are (2100±500) meV in the low-temperature regime I and (60±10) meV in the high-temperature state II.


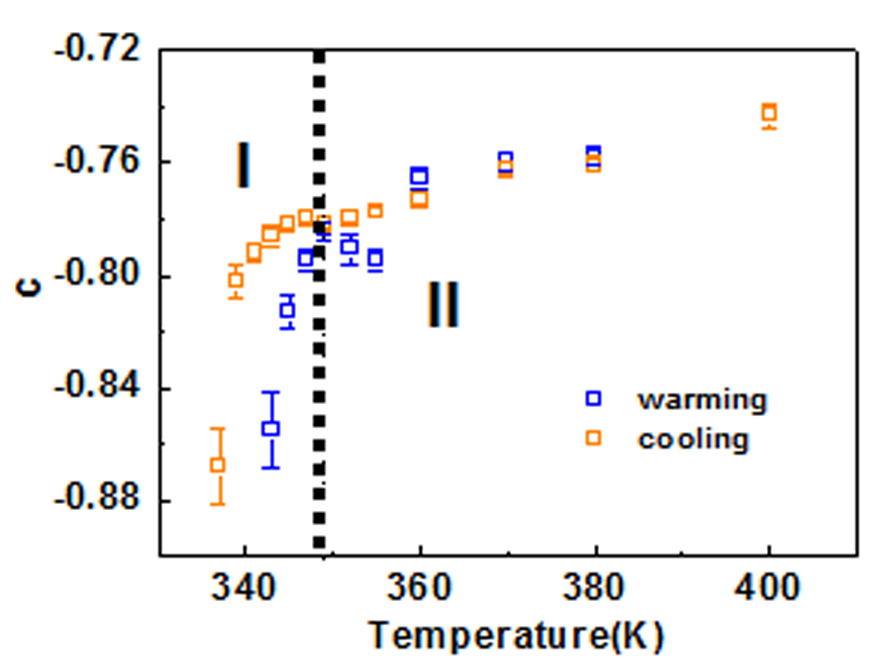


Supplementary Figure 7: Hysteresis loop of the Drude-Smith c parameter of VO_2_(*M*1) grown on SLAO as obtained from the fitting in Figure S5. In the conductivity state II, the value of *c* is almost temperature independent and similar for the both the warming and the cooling cycles. However, in the conductivity state I, there is strong hysteresis as well as strong temperature dependence. This behaviour of *c* is a signature of first order phase transition through an intermediate percolating network where the increase in temperature favors the growth of metallic domains.


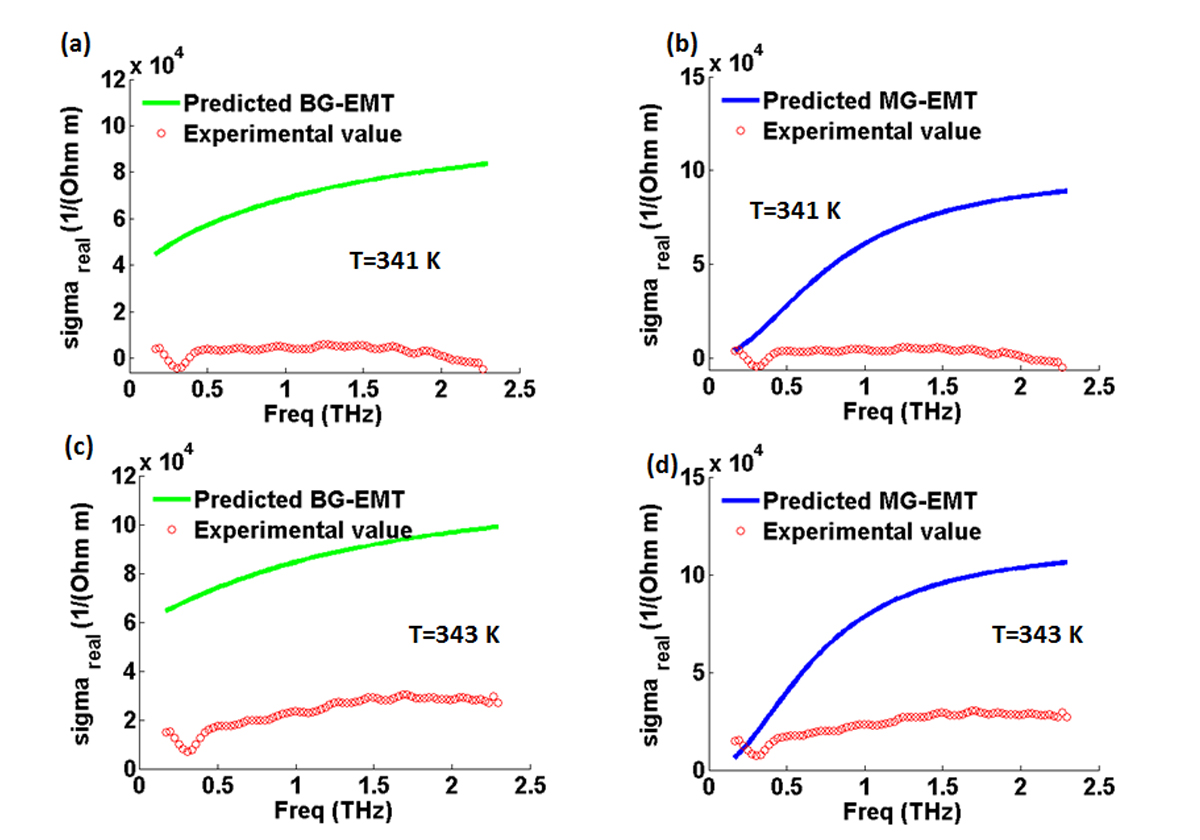


Supplementary Figure 8: Comparision of the experimental values of
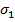
 for VO_2_(*M*1)with the predicted values of conductivities using the effective medium theories during the transition for temperatures 341 K(a, b) and 343 K(c,d). BG-EMT stands for the Bruggeman effective medium theory and MG-EMT stands for Maxwell Garnett effective medium theory. The equations used for the BG-EMT model is


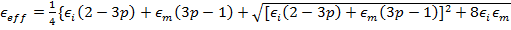


where
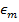
 and
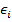
 are the dielectric functions of the metallic regions and the insulating regions respectively. The volume fraction of the conducting region is represented by the volume fraction *p.* For the insulating phase
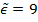
 while for the metallic phase it is given by the Drude model
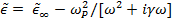
. Similar to the work by P.Mandal *et.al.*, we used the standard values of
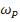
 and
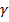
 to determine the dielectrinc function in the metallic phase.[^3^](#_ENREF_3)

The equation used for the MG-EMT model is [^1^](#_ENREF_1)


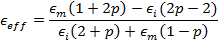


The fractional metallic part *p* is given by the formula[^1^](#_ENREF_1)


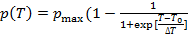
)

*p*_max_=0.95, *T*_0_=341 K and
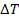
 =6 K was used for our VO_2_(*M*1) sample. [^1^](#_ENREF_1)


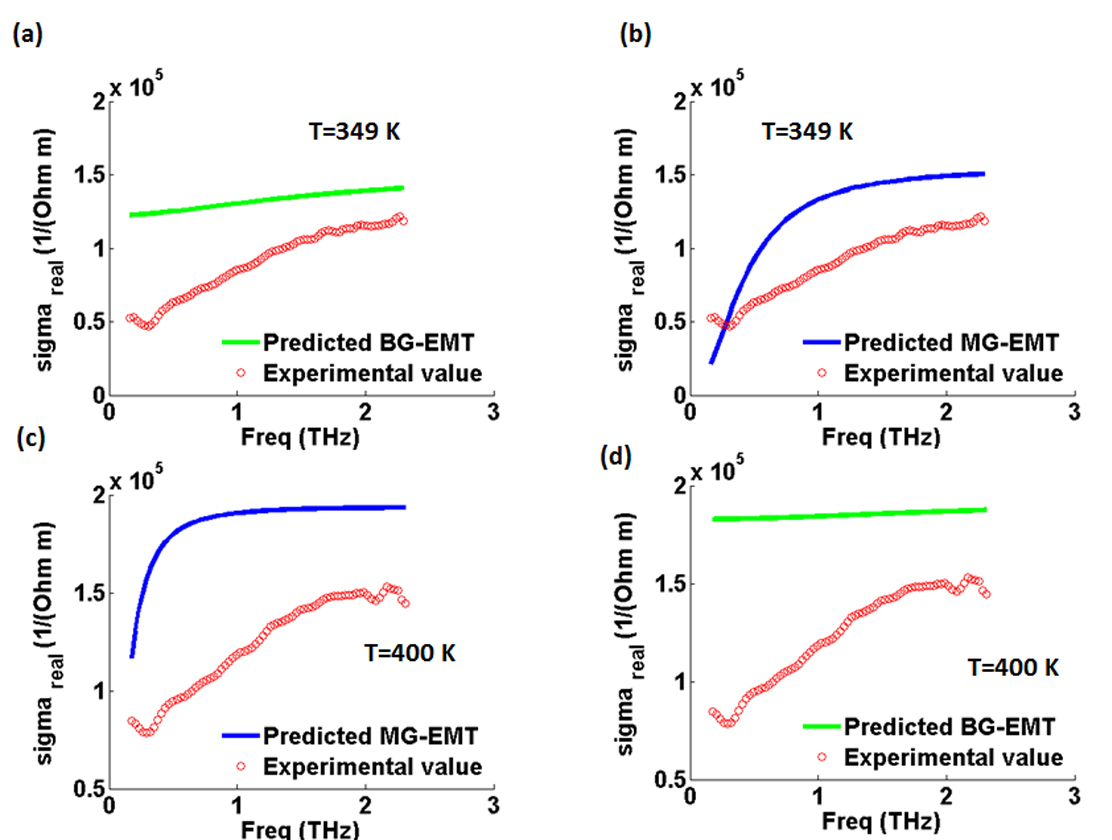


Supplementary Figure 9: Comparision of the experimental values of
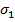
 for VO_2_(*R*) (after the structural transformation in the *R* phase) with the effective medium theories for temperature 349 K(a,b) and 400 K(c,d)

1 Jepsen, P. *et al.* Metal-insulator phase transition in a VO_2_ thin film observed with terahertz spectroscopy. *Phys. Rev. B* **74**, 205103, (2006).

2 Cocker, T. L. *et al.* Terahertz conductivity of the metal-insulator transition in a nanogranular VO_2_ film. *Appl. Phys. Lett.* **97**, 221905, (2010).

3 Mandal, P., Speck, A., Ko, C. & Ramanathan, S. Terahertz spectroscopy studies on epitaxial vanadium dioxide thin films across the metal-insulator transition. *Opt. Lett.* **36**, 1927, (2011).
